# Does basic information concerning nutrition improve the information needs of breast cancer patients? An evaluation

**DOI:** 10.1007/s00520-020-05385-1

**Published:** 2020-03-07

**Authors:** Sophie E. Groß, Doreen Weidner, Natalia Cecon, Holger Pfaff, Carmen Strauch, Nadine Scholten

**Affiliations:** 1 Institute of Medical Sociology, Health Services Research and Rehabilitation Science (IMVR), Faculty of Human Sciences and Faculty of Medicine, University of Cologne, Eupener Straße 129, 50933 Cologne, Germany; 2 LVR-Institute of Health Care Research, LVR Clinic Cologne, Wilhelm-Griesinger Str. 23, 51109 Cologne, Germany; 3 Department of Integrated Oncology, Center of Integrated Oncology (CIO) Aachen, Bonn, Cologne, Düsseldorf (ABCD), University Hospital of Bonn, Venusberg-Campus 1, 53127 Bonn, Germany

**Keywords:** Fact sheet, Breast cancer patient, Information need, Nutrition, Intervention, Health services research

## Abstract

**Purpose:**

International and national studies have shown unmet information needs regarding nutrition in breast cancer patients. An intervention study has examined the question of the extent to which a fact sheet on the topic of nutrition is suitable to cover the need for information of breast cancer patients.

**Method:**

The fact sheet with basic information on nutrition was distributed in 21 intervention breast care centres in 2017. The use of the fact sheets was evaluated in a quasi-experimental design as part of the annual breast cancer patients’ survey of the University of Cologne. The breast cancer patients considered were being treated with primary breast carcinoma in a hospital in North Rhine-Westphalia. A multilevel analysis was carried out in order to quantify the effect of the intervention.

**Results:**

Unmet information needs are experienced more by younger and non-native German-speaking patients. With regard to education, patients without a graduation and a high grade of education express more unmet information needs. The multilevel analysis showed that patients who were treated at an intervention site and therefore possibly received the fact sheet have a significantly higher chance of their information needs being met (*OR* = 1.45; *p* ≤ 0.05).

**Conclusion:**

The intervention study showed that a fact sheet with basic information on nutrition is a possible instrument to satisfy the information needs of breast cancer patients and therefore reduce unmet information needs regarding nutrition. This intervention study is a pragmatic example on how to reduce unmet information needs among breast cancer patients in Germany.

## Introduction

Following Schlegel et al., patients’ information needs are defined as a conscious expression, which can be verbal or nonverbal, of a desire for knowledge to answer clinical questions within patient care [1]. Understanding and meeting the information needs of patients is crucial to improving their quality of care, especially in the context of the information needs of patients with life-threating diagnoses, such as cancer. Most cancer patients’ information needs are concerned with the likelihood of a cure, the cancer stage and prognosis, treatment options, therapy, side effects, logistics, and the family risk of developing cancer [2, 3].

Unmet information needs are experienced by many different cancer patients in all stages, even though certain demographic and clinical characteristics influence patients’ prioritization of information needs [3–6]. Various differences have been reported, such as those between religious beliefs, age, whether they care for themselves alone, household income, educational level, time since cancer diagnosis, differences between men and women, and different needs with regard to the form of the tumour and the course of the disease [2–4]. This is particularly relevant when taking into consideration that there seems to be a link between unmet information needs and quality of life [7].

With 69,000 women being diagnosed with breast cancer every year, breast cancer is the most common cancer for women in Germany and was the most common cause of cancer-related deaths for women in 2016 worldwide [8, 9]. Unmet information needs that are experienced by breast cancer patients primarily focus on things that the patient can do to recover, as well as issues of fertility and sexuality, cancer spreading or chances of a cure, future thoughts, side effects and medication, health promotion, medical examination results, and treatment options [10–13]. The information needs of breast cancer patients differ depending on education, cultural factors, native language, and place of residence (rural or urban settings) [3, 14, 15]. There are also differences with regard to age in terms of information needs. Young breast cancer patients in particular face unmet information needs, regarding issues of fertility and family planning, as well as suffering from sexual dysfunction and menopausal-related concerns regarding adjuvant therapy [16–18].

Above all, breast cancer patients want to know what they can contribute to their recovery process in terms of survivorship, which means information needs of the patients concern the modification of their lifestyle regarding nutrition, exercise, and breast self-examination [19].

The fact that there is a need for more knowledge about healthy nutrition which is often not satisfied has already been demonstrated in the context of a survey with breast cancer patients in Japan [7]. Similar results come from the annual breast cancer patient survey in North Rhine-Westphalia, Germany. The 2016 survey of 4489 breast cancer patients showed that 39.9% of breast cancer patients would have liked to receive more information about nutrition [20].

The aim of this intervention study is to show if the information need concerning nutrition for breast cancer patients in Germany can be met with the distribution of fact sheets regarding basic information on nutrition.

## Materials and methods

Every year, the Institute for Medical Sociology, Health Services Research, and Rehabilitation Science (IMVR) of the University of Cologne surveys breast cancer patients with a primary breast carcinoma who have undergone surgery at a breast cancer centre in North Rhine-Westphalia [21]. This intervention study focuses on the results of the surveys conducted in 2016 and 2017 and includes only data from hospitals that participated in both survey periods (*n* = 86 breast cancer centres). The Cologne Patients Questionnaire for Breast Cancer (CPQ-BC) addresses the specific needs of breast cancer patients regarding the subjective perception of the treatment, hospital organization, communication, interaction with staff, and the course of the discharge from hospital [21].

As an intervention to address the unmet information need concerning nutrition, a two-page fact sheet was developed in cooperation with the Centre of Integrative Oncology in Cologne. This fact sheet summarizes basic information about nutrition, including the ten rules for a healthy diet from the German Nutrition Society (DGE) and was developed on the basis of current study results [22]. It does not focus on a diet that is adjusted especially for breast cancer patients. It is written in German in simple terms and does not include technical terminology.

**Fig. 1:**
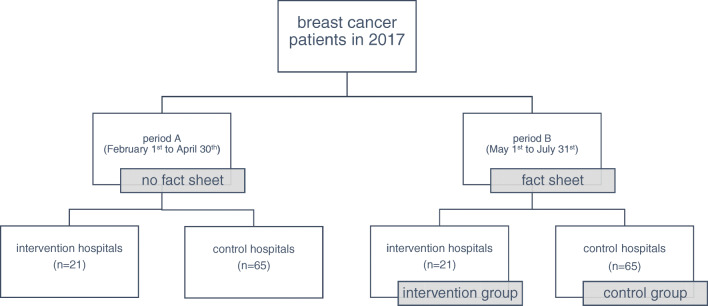
Quasi experimental study design of the intervention study

The survey period of 2017, which is from 01 February to 31 July, was divided into two periods: period A with no fact sheet available (from 01 February to 30 April = control period); and period B, where the fact sheet has been distributed in the 21 intervention hospitals (01 May to 31 July = intervention period; please see Fig. 1 Quasi-experimental study design of the intervention study). In the following, patients who were treated in an intervention breast cancer centre during period B, where the fact sheets have been distributed, are called intervention group. This allows for the comparison of patient groups that were treated in the same hospitals in 2017 who potentially did or did not receive the fact sheet. This intervention study, embedded in a quasi-experimental design, can reduce the effect of variations, such as employee changes, management restructuring, or clinic reformations, as each hospital can be compared to itself within the same year.

The information need of female breast cancer patients was measured with the dichotomized item “Would you have liked to receive more information regarding nutrition from your Breast Centre?”. The answers from female breast cancer patients in 2016 (*n* = 4489) and 2017 (*n* = 4626), being treated in the same hospitals, were compared. Furthermore, self-reported sociodemographic information, such as age, highest graduation certificate achieved, and native language, were recorded and related to the information needs experienced by the women. Only women were included in the intervention study, as the number of cases of male breast cancer patients in the sample is too small to make a reliable statement about gender-specific differences in information needs.

SPSS Statistics V25 was used to descriptively analyse the frequencies of women experiencing an unmet information need regarding nutrition. Depending on the scale levels, intercorrelations were checked by calculating Pearson’s or Spearman’s correlation coefficients and chi-square tests. Cases with missing data for the dependent and independent variables were excluded (listwise deletion). Using Stata version MP15, a multilevel analysis was carried out in order to explain the association between sociodemographic characteristics and the information need experienced, taking into account the clustering of patients in the individual breast cancer centres. The null model was calculated in order to have a reference on how much of the explanation of variance in information need is attributed to the hospitals themselves and not to the patient’s characteristics. Model I explains the variance in information need under control of patients’ characteristics, such as age, education, and native language. The full model explains the variance in information need under the control of the intervention variable (fact sheet possibly received). In model I and in the full model, only patients who were treated during the intervention period were considered in the analysis.

## Results

### Descriptive results

The response rate for the returned questionnaires in 2017 was 89% and 86.9% in 2016. The demographic characteristics of the 4626 breast cancer patients participating in the annual breast cancer survey in 2017 are shown in Table 1. The median age of the sample is 61 years (minimum age 24 years, maximum age 97 years). 28.1% of the breast cancer patients achieved a graduation certificate at junior high school, 23.0% achieved a graduation certificate at a lower secondary school, and 18.7% achieved A-Levels. 92.3% of the patients are native German speakers. There are no identifiable group differences (chi-square tests) when comparing patients’ demographic characteristics in the intervention group to the overall data from 2017 concerning age (*p* = 0.41), highest graduation certificate achieved (*p* = 0.467), or native language (*p* = 0.097). In conclusion, the intervention group does not significantly differ from the whole sample of 2017. Comparing the unmet information need of female breast cancer patients, there are no noteworthy differences in 2016 and 2017 (39.9% in 2016 and 39.4% in 2017).

**Table 1:** The demographic characteristics of 4626 female breast cancer patients who were included in the intervention study are shown

	Sample (overall data from 2017 (*n*)	Intervention group (possibly receiving the fact sheet) (*n*)	Control group (not receiving the fact sheet) (*n*)
Age			
*Median*	61 years	61 years	60 years
*18–29 years*	0.5% (21)	0.9% (5)	0.5% (8)
*30–39 years*	3.1% (144)	4.3% (24)	3.4% (58)
*40–49 years*	13.3% (608)	13.7% (76)	13.2% (225)
*50–59 years*	28.9% (1324)	29.7% (165)	28.2% (482)
*60–69 years*	28.0% (1283)	26.8% (149)	27.7% (474)
*70–79 years*	18.8% (863)	17.6% (98)	18.9% (323)
*80 years or older*	7.5% (342)	7.0% (39)	8.2% (141)
*Total*	100% (4585)	100%^a^ (556)^a^	100% (1711)
Highest graduation certificate achieved			
*Without school graduation certificate*	2.0% (90)	2.4% (13)	1.4% (24)
*Lower secondary school*	23.0% (1035)	20.6% (113)	24.3% (406)
*Intermediate secondary school*	16.3% (733)	16.1% (88)	14.8% (248)
*Junior high school*	28.1 (1263)	27.0% (148)	28.1 (471)
*Upper secondary school*	11.9% (533)	13.3% (73)	11.5% (193)
*A-levels*	18.7% (842)	20.6% (113)	19.8% (332)
*Total*	100% (4496)	100%^b^ (548)^b^	100% (1711)
Native Language			
*German*	92.3% (4179)	94.0% (519)	92.6% (1565)
*Another native language*	7.7% (351)	6.0% (33)	7.4% (125)
*Total*	100% (4530)	100%^c^ (552)^c^	100% (1711)

Comparing the overall data from 2017 (sample) to the intervention and control groups

^a^Group differences comparing the intervention group with the overall data of 2017: Chi-square test (Pearson) *p* = 0.41

^b^Group differences comparing the intervention group with the overall data of 2017: Chi-square test (Pearson) *p* = 0.467

^c^Group differences comparing the intervention group with the overall data of 2017: Chi-square test (Pearson) *p* = 0.097

The unmet information need regarding nutrition is age related, as can be seen in Table 2, wherein younger breast cancer patients experience higher information needs. The highest unmet information need can be found in the age group of 30 to 39 years (50%). Breast cancer patients that achieved a high grade of education as well as patients without a graduation certificate experience a higher unmet information need (Table 3). Native German speakers experience smaller information needs regarding nutrition than non-native German speakers (Table 4).

**Table 2:** Comparing the overall data from 2017 to the intervention group and the control group in 2017 regarding information need experienced

	Information need?	18–29 years % (*n*)	30–39 years % (*n*)	40–49 years % (*n*)	50–59 years % (*n*)	60–69 years % (*n*)	70–79 years % (*n*)	80+ years % (*n*)	Missing age group (*n*)	All age groups % (*n*)	Total % (n)
Overall data in 2017	Yes	42.9% (9)	50.0% (72)	44.6% (269)	43.5% (568)	36.5% (456)	35.2% (284)	29.3% (90)	- (3)	39.4%^a^ (1748)	100% (1751)
	No	57.1% (12)	50.0% (72)	55.4% (334)	56.5% (739)	63.5% (792)	64.8% (522)	70.7% (217)	- (1)	60.6% (2688)	100% (2689)
Intervention group (possibly receiving the fact sheet)	Yes	20.0% (1) 80.0%	29.2% (7) 70.8%	32.9% (25) 67.1%	39.6% (65) 60.4%	25.7% (37) 74.3%	29.7% (27) 70.3%	24.2% (8) 75.8%	- (0) -	31,7%^b^ (170) 68.3%	100% (170) 100%
	No	(4)	(17)	(51)	(99)	(107)	(64)	(25)	(1)	(367)	(368)
Control group (no fact sheet)	Yes	50.0% (4)	60.3% (35)	46.6% (103)	42.5% (203)	35.9% (165)	36.7% (110)	31.0% (40)	- (−)	39.9%^c^ (660)	100% (660)
	No	50.0% (4)	39.7% (23)	53.4% (118)	57.5% (275)	64.1% (295)	63.3% (190)	69.0% (89)	- (−)	60.1% (994)	100% (994)

Looking at female breast cancer patients; separated into seven age groups

^a^Group differences within the overall data from 2017: Chi-square test (Pearson) *p* = 0.000

^b^Group differences within the intervention group (possibly receiving the fact sheet): Chi-square test (Pearson) *p* = 0.195

^c^Group differences within the control group (no fact sheet): Chi-square test (Pearson) *p* = 0.000

**Table 3:** Comparing the overall data from 2017 to the intervention group and the control group in 2017 regarding information need experienced

	Information need?	Without school graduation certificate % (*n*)	Lower secondary school % (*n*)	Intermediate secondary school % (*n*)	Junior high school % (*n*)	Upper secondary school % (*n*)	A-levels % (*n*)	Missing highest graduation certificate achieved (*n*)	All groups % (*n*)	Total % (*n*)
Overall data in 2017	Yes	41.6% (37)	32.4% (314)	37.3% (265)	40.0% (494)	47.1% (248)	43.8% (363)	- (30)	39.5%^a^ (1721)	100% (1751)
	No	58.4% (52)	67.6% (655)	62.7% (446)	60.0% (741)	52.9% (278)	56.2% (466)	- (51)	60.5% (2638)	100% (2689)
Intervention group (possibly receiving the fact sheet)	Yes	46.2% (6)	24.0% (25)	34.5% (29)	33.6% (49)	31.5% (23)	33.6% (37)	- (1)	31.9%^b^ (169)	100%** (170)
	No	53.8% (7)	76.0% (79)	65.5% (55)	66.4% (97)	68.5% (50)	66.4% (73)	- (7)	69.1% (361)	100% (368)
Control group (no fact sheet)	Yes	37.5% (9)	35.4% (135)	38.0% (93)	40.5% (185)	49.5% (94)	40.8% (133)	- (11)	39.9%^c^ (649)	100% (660)
	No	62.5% (15)	64.6% (246)	62.0% (152)	59.5% (272)	50.5% (96)	59.2% (193)	- (20)	60.1% (974)	100% (994)

Looking at female breast cancer patients, separated into groups according to the highest graduation certificate achieved

^a^Group differences within the overall data from 2017: Chi-square test (Pearson) *p* = 0.000

^b^Group differences within the intervention group after May (possibly receiving the fact sheet): Chi-square test (Pearson) *p* = 0.443

^c^Group differences within the control group (no fact sheet): Chi-square test (Pearson) *p* = 0.051

**Table 4:** Comparing the overall data from 2017 to the intervention group and the control group in 2017 regarding information need experienced

	Information need?	Native speaker (German) % (*n*)	Non-native German speaker % (*n*)	Missing native language (*n*)	All groups % (*n*)	Total % (*n*)
Overall data in 2017	Yes	38.6% (1560)	49.9% (171)	- (20)	39.5%^a^ (1731)	100% (1751)
	No	61.4% (2484)	50.1% (172)	- (33)	60.5% (2656)	100% (2689)
Intervention group (possibly receiving the fact sheet)	Yes	31.6% (158)	36.4% (12)	- (5)	31.9%^b^ (170)	100% (175)
	No	68.4% (342)	63.6% (21)	- (2)	68.1% (363)	100% (365)
Control group (no fact sheet)	Yes	38.9% (589)	50.0% (61)	- (10)	39.8%^c^ (650)	100% (660)
	No	61.1% (924)	50.0% (61)	- (9)	60.2% (985)	100% (994)

Looking at female breast cancer patients, separated into groups according to the patient’s native language

^a^Group differences within the overall data of 2017: Chi-square test (Pearson) *p* = 0.000

^b^Group differences within the intervention group (possibly received the fact sheet): Chi-square test (Pearson) *p* = 0.570

^c^Group differences within the control group (no fact sheet): Chi-square test (Pearson) *p* = 0.016

### Intervention

The intervention reduced the unmet information need comparing the control group to the intervention group on a statistically significant level (*p* ≤ 0.01, chi-square test), from 39.9% to 31.6%.

Looking at the patients within the intervention group with unmet information needs versus patients with met information needs, no significant differences (chi-square tests) regarding age (*p* = 0.195), education (*p* = 0.443), and native language (*p* = 0.570) can be seen. Comparing the intervention and the control groups, the highest reduction in information need can be seen in patients with higher educational levels (from 40.8% to 33.6% in patients that achieved A-levels), lower age (from 60.3% to 29.2% in patients aged 30 to 39 years), and non-native German speakers (from 50.0% to 36.4% in non-native German speakers), as can be seen in Tables 2, 3, and 4.

### Multilevel analysis

As the data were structured hierarchically with patients nested within hospitals, multilevel modelling was used to account for clustering [23]. First, a two-level model without predictors (null model) was fitted in order to calculate the intraclass correlation coefficient (ICC). The ICC provides insights into the degree to which the dependent variable (information need) varies between hospitals. In a second step, patient characteristics such as age, education, and native language were added as predictors at the patient’s level (model I). In the full model, the intervention variable (fact sheet possibly received) was added as a predictor at the patient’s level.

In bivariate chi-square tests, significant relationships between unmet information needs regarding nutrition and the patient’s characteristics native language, education, and age could be shown (Table 2, 3, and 4). In the multilevel analysis in model I, the sociodemographic characteristics education and native language were no longer significantly associated with the unmet information need regarding nutrition (Table 5). With the addition of the intervention variable (fact sheet possibly received) in the full model in the multilevel analysis, the age of the breast cancer patient remains a significant sociodemographic factor influencing the unmet information need regarding nutrition (Table 5).

**Table 5:** Results from the multilevel logistic regression analysis; odds ratios (95% confidence intervals)

	Null model	Model I	Full model
Fact sheet possibly received	**–**	**–**	**1.45 (1.09–1.92)**
*Ref. no fact sheet received*			
Age	**–**	**1.02 (1.01–1.02)**	**1.02 (1.01–1.02)**
Highest graduation certificate achieved			
Without school graduation certificate	–	0.93 (0.46–1.88)	0.85 (0.42–1.73)
Lower secondary school	–	1.04 (0.76–1.42)	1.03 (0.75–1.41)
Intermediate secondary school	**–**	1.06 (0.78–1.44)	1.04 (0.77–1.41)
Junior high school	–	0.99 (0.77–1.28)	0.97 (0.75–1.26)
Upper secondary school *Ref. Abitur*	–	0.80 (0.59–1.06)	0.79 (0.57–1.08)
Native language German *Ref. foreign speaker*		0.75 (0.53–1.06)	0.75 (0.53–1.05)
*n* patient	2192	2181	2168
*n* hospitals	86	84	84
ICC	0.04	0.04	0.03
Random-effects parameters hospital level estimate (SE)	0.35 (0.07)	0.35 (0.05)	0.32 (0.08)

Taking into account female breast cancer patients that were treated in the intervention period

statistically significant odds ratios are in boldface; Ref., reference category; ICC, Intraclass correlation coefficient; SE, standard error

Patients that possibly received the fact sheet have a significantly higher chance of a met information need (*OR* = 1.45; *p* ≤ 0.05) under control of sociodemographic variables and when taking the hierarchically data structure into account. Even after differences at hospital level and variables at the patient level as education and native language (model I) and the intervention variable (fact sheet possibly received) (full model) have been taken into account, patients who have a higher age have a significantly higher chance of a met information need (*OR* = 1.02; *p* ≤ 0.05),

Due to listwise deletion, the ‘null model’ consists of data from 2270 female breast cancer patients clustered in 84 hospitals. Model I consists of data of 2181 female breast cancer patients, and the ‘full model’ consists of data from 2168 female breast cancer patients clustered in 84, by reason of listwise deletion because of missing values on the patient level. Four percent (*ICC* = 0.04) of the explanation of variance in information need can be attributed to characteristics of the treating hospitals.

## Discussion

Possibly receiving the fact sheet with basic nutritional information leads to a significant reduction in information needs regarding nutrition experienced by breast cancer patients who are being treated in a breast cancer centre in North Rhine-Westphalia. This intervention study shows a significant reduction by 8.3 percentage points from 39.9 to 31.7% with regard to this unmet information need. Descriptive statistics show a greater reduction in unmet information need in the intervention group among younger patients. This result could not be confirmed under the controls of education and native language and the intervention variable (fact sheet possibly received) in the full model of the multilevel analysis: In general, older breast cancer patients experience a lower information need regarding nutrition, independently of the intervention. Older cancer patients tend to search less for information themselves and to behave more passively in the time period following treatment [24].

The multilevel analysis confirmed the results of the bivariate chi-square tests and showed that patients possibly receiving the fact sheet have a significantly higher chance of a met information need, even after the hierarchical data structure has been taken into account and even after controlling for co-variables at the patients level (age, education, and native language). However, the descriptive results show that the highest reduction of information need was in patients with higher educational levels and non-native German speakers. The cross-sectional study conducted by Ellegaard at al. report that one of the frequently experienced information needs among women is the need to receive understandable and up-to-date information [25]. In the qualitative study of Kwok et al., perceptions of information needs and social support among women were surveyed, showing that a clear desire of linguistically appropriate information was expressed by the majority of breast cancer patients [14]. The results of this intervention study, as well as the results from the surveys mentioned, emphasize the importance of a clearly structured and simply written informational tool, such as a fact sheet.

A covered information need can be associated with patient reported outcomes. A cross-sectional descriptive study from Miyashita et al., analysing unmet information needs and QOL, was able to show that unmet information needs experienced by young breast cancer survivors are directly linked to QOL by comparing the satisfaction of breast cancer patients with the information received from the hospitals to their results in the QOL subscales [7]. There are international studies trying to decrease a general unmet information need not focusing on nutrition, reduce distress in cancer patients, and increase the quality of life (QOL) of breast cancer patients, using purpose-built, information-based websites or web-based self-management programmes, with inconsistent results [26, 27]. White et al. are trying to ascertain whether access to an information-based website significantly reduces the distress in younger women being diagnosed with breast cancer [26]. As the information needs of women vary at different stages of the treatment, the website was clustered into sections that focus separately on diagnosis, treatment, and survivorship. They were able to demonstrate the unmet supportive care needs experienced by the women included in the study, but could not show that access to an information-based website reduces distress in younger women or that it significantly decreases their information and supportive care needs [26]. The multicentre, randomized controlled, parallel-group trial by Van den Berg et al., examining a web-based self-management programme, which has a duration of 16 weeks, showed a significant reduction of distress in breast cancer patients [27]. This shows how important it is to cover information needs in order to positively influence the quality of life of patients.

QOL is affected more negatively in younger women with breast cancer than in women without breast cancer by factors such as recurrence, relationship problems, problems with sexual function, and fewer coping strategies [28]. What needs to be borne in mind when comparing the intervention study that was conducted by the IMVR to the studies mentioned above is that this intervention study has a clear focus only on meeting the information needs of breast cancer patients concerning nutrition. It does not take into account patient-reported outcomes in terms of QOL or distress of breast cancer patients. Furthermore, the fact sheet was distributed systematically in about 25% (*n* = 21) of the breast cancer centres participating in the annual survey, where the patients were treated for primary breast cancer after the diagnosis, and the study does not differentiate between different stages of breast cancer diagnosis and treatment.

## Limitations

Due to organizational processes in the breast cancer centres, the distribution of the fact sheet could not be randomized on the patient’s level. The intervention houses were not randomized either but voluntarily agreed to distribute the fact sheets. Despite the high response rate, participation bias cannot be ruled out, as patients with disabilities indicated more often that they had received assistance in completing the questionnaire. When patient-reported data are assessed as in the present study, there is a risk of social desirability bias and common method bias. Taking into consideration the expected bias of the data, a quasi-experimental study design was applied by distributing the fact sheet only in the second survey period of 2017 (01 May to 31 July), leading to an elimination of variations such as employee changes, management restructuring, or clinic reformations. Also, for this reason, hierarchical multilevel models were used to correct for effects at the hospital level. Furthermore, it is not possible to ensure that all women being treated in the intervention group actually received the fact sheet, because the intervention hospitals themselves were responsible for the distribution of the fact sheet among the breast cancer patients. For this reason, however, it can be assumed that even greater effects could be achieved if the actual receipt of the fact sheets can be taken into account.

## Conclusion

The analysis showed that possibly receiving the fact sheet with basic nutritional information leads to a significantly higher chance of information needs being met in breast cancer patients being treated in a breast care centre in North Rhine-Westphalia. A higher age correlates with a higher chance of an information need being met, as shown in the multilevel analysis. With regard to patient education and native language, no significant differences could be found in the multilevel models. This indicates that a fact sheet, when written simply and in easy-to-understand terms, can be a useful tool, which seems to be effective in reducing unmet information needs.

The aim of the study – to show that a fact sheet with basic nutritional information reduces the unmet information needs of breast cancer patients in Germany – was achieved. The results show that a fact sheet is a feasible instrument to reduce information needs significantly. This pragmatic intervention study design can also be applied to other topics of unmet information needs, aside from nutrition.

Furthermore, this study is – to our knowledge – the first intervention study trying to decrease the unmet information needs in breast cancer patients using a fact sheet.

## References

[1] Schlegel CS, Yoder LH, Jones TL (2019) Clinical information needs: a concept analysis. ANS Adv Nurs Sci. 10.1097/ANS.0000000000000260 10.1097/ANS.0000000000000260 31299685

[2] Ahamad A, Wallner P, Salenius S, Ross R, Fernandez E (2019). Information needs expressed during patient-oriented oncology consultations: quantity, variation, and barriers. J Cancer Educ.

[3] Bei AW, Lai MT, Choi KC, Li PW, So WK (2015). Factors in the prioritization of information needs among Hong Kong Chinese breast cancer patients. Asia Pac J Oncol Nurs.

[4] Faller H, Koch U, Brahler E, Harter M, Keller M, Schulz H, Wegscheider K, Weis J, Boehncke A, Hund B, Reuter K, Richard M, Sehner S, Szalai C, Wittchen HU, Mehnert A (2016). Satisfaction with information and unmet information needs in men and women with cancer. J Cancer Surviv.

[5] Matsuyama RK, Kuhn LA, Molisani A, Wilson-Genderson MC (2013). Cancer patients’ information needs the first nine months after diagnosis. Patient Educ Couns.

[6] Rha SY, Lee HJ, Lee J (2019). Unmet needs in the physical and daily living domain mediates the influence of symptom experience on the quality of life of gastric cancer patients. Support Care Cancer.

[7] Miyashita M, Ohno S, Kataoka A, Tokunaga E, Masuda N, Shien T, Kawabata K, Takahashi M (2015). Unmet information needs and quality of life in young breast cancer survivors in Japan. Cancer Nurs.

[8] Fitzmaurice C, Akinyemiju TF, Al Lami FH, Alam T, Alizadeh-Navaei R, Allen C (2018). Global, regional, and national cancer incidence, mortality, years of life lost, years lived with disability, and disability-adjusted life-years for 29 cancer groups, 1990 to 2016: a systematic analysis for the global burden of disease study. JAMA Oncology.

[9] Robert Koch-institut (Editor), Gesellschaft der epidemiologischen Krebsregister in Deutschland e.V. (Editor) (2017) Krebs in Deutschland für 2013/2014. Krebs in Deutschland, vol 11. https://www.krebsdaten.de/Krebs/DE/Content/Publikationen/Krebs_in_Deutschland/krebs_in_deutschland_node.html

[10] Cheng KK, Darshini Devi R, Wong WH, Koh C (2014). Perceived symptoms and the supportive care needs of breast cancer survivors six months to five years post-treatment period. Eur J Oncol Nurs.

[11] Raupach JC, Hiller JE (2002). Information and support for women following the primary treatment of breast cancer. Health Expect.

[12] Charif AB, Bouhnik AD, Rey D, Provansal M, Courbiere B, Spire B, Mancini J (2015). Satisfaction with fertility- and sexuality-related information in young women with breast cancer--ELIPPSE40 cohort. BMC Cancer.

[13] Halbach SM, Ernstmann N, Kowalski C, Pfaff H, Pförtner TK, Wesselmann S, Enders A (2016). Unmet information needs and limited health literacy in newly diagnosed breast cancer patients over the course of cancer treatment. Patient Educ Couns.

[14] Kwok C, White K (2014). Perceived information needs and social support of Chinese-Australian breast cancer survivors. Support Care Cancer.

[15] Wang S, Li Y, Li C, Qiao Y, He S (2018). Distribution and determinants of unmet need for supportive care among women with breast cancer in China. Med Sci Monit.

[16] Harirchi I, Montazeri A, Zamani Bidokhti F, Mamishi N, Zendehdel K (2012). Sexual function in breast cancer patients: a prospective study from Iran. J Exp Clin Cancer Res.

[17] Thewes B, Meiser B, Taylor A, Phillips KA, Pendlebury S, Capp A, Dalley D, Goldstein D, Baber R, Friedlander ML (2005). Fertility- and menopause-related information needs of younger women with a diagnosis of early breast cancer. J Clin Oncol.

[18] Braun LA, Zomorodbakhsch B, Keinki C, Huebner J (2019). Information needs, communication and usage of social media by cancer patients and their relatives. J Cancer Res Clin Oncol.

[19] Cheng KKF, Cheng HL, Wong WH, Koh C (2018). A mixed-methods study to explore the supportive care needs of breast cancer survivors. Psychooncology.

[20] Groß SE, Weidner D, Pfaff H, Scholten N (2018). Interventionsstudie zum Thema Ernährung bei Brustkrebs. Senologie - Zeitschrift für Mammadiagnostik und -therapie.

[21] Ansmann L, Kowalski C, Pfaff H (2016). Ten years of patient surveys in accredited breast centers in North Rhine-Westphalia. Geburtshilfe Frauenheilkunde.

[22] Deutsche Gesellschaft für Ernährung e.V. (2019) Vollwertig essen und trinken nach den 10 Regeln der DGE. Deutsche Gesellschaft für Ernährung e.V. https://www.dge.de/ernaehrungspraxis/vollwertige-ernaehrung/10-regeln-der-dge/. Accessed 02.10. 2019

[23] Bender R, Lange S (2001). Adjusting for multiple testing--when and how?. J Clin Epidemiol.

[24] Eheman C, Berkowitz Z, Lee JW, Mohile SG, Purnell JQ, Roscoe JA, Johnson DB, Kirshner JJ, Morrow GR (2009). Information-seeking styles among cancer patients before and after treatment by demographics and use of information sources. J Health Commun.

[25] Ellegaard MB, Grau C, Zachariae R, Bonde Jensen A (2017). Fear of cancer recurrence and unmet needs among breast cancer survivors in the first five years. A cross-sectional study. Acta Oncol.

[26] White V, Farrelly A, Pitcher M, Hill D (2018). Does access to an information-based, breast cancer specific website help to reduce distress in young women with breast cancer? Results from a randomised trial. Eur J Cancer Care.

[27] van den Berg SW, Gielissen MFM, Custers JAE, van der Graaf WTA, Ottevanger PB, Prins JB (2015). BREATH: web-based self-management for psychological adjustment after primary breast cancer--results of a multicenter randomized controlled trial. J Clin Oncol.

[28] Avis NE, Crawford S, Manuel J (2005). Quality of life among younger women with breast cancer. J Clin Oncol.

